# Supplementary material for: Does the Acknowledgement of αS1-Casein Genotype Affect the Estimation of Genetic Parameters and Prediction of Breeding Values for Milk Yield and Composition Quality-Related Traits in Murciano-Granadina?
Source: Animals (Basel). 2019 Sep 13;9(9):679. doi: 10.3390/ani9090679 (PMC6770805; doi:10.3390/ani9090679)
Supplement: Supplementary file 1 [file animals-09-00679-s001.zip › Supplementary Table S1.docx]

**Does the acknowledgement of αS1-casein genotype affect the estimation of genetic parameters and prediction of breeding values for milk yield and composition quality-related traits in Murciano-Granadina?**

**María Gabriela Pizarro Inostroza, Vincenzo Landi, Francisco Javier Navas González, Jose Manuel León Jurado, Amparo Martínez Martínez, Javier Fernández Álvarez and Juan Vicente Delgado Bermejo**

**Supplementary Table S1.** *Number of observations and goats sorted per αS1-casein (CSN1S1) genotype.*

| Genotype | Number of observations | Number of goats | Sample frequency | Caravaca et al. (2009)^a^  (n=509 goats) |
| --- | --- | --- | --- | --- |
| AA | 72 | 25 | 3.52% | 3.30% |
| AB | 25 | 10 | 1.41% | 5.60% |
| BB | 24 | 10 | 1.41% | 13.9% |
| AE | 515 | 180 | 25.35% | 9.60% |
| BE | 1416 | 473 | 66.62% | 30.00% |
| EF | 38 | 12 | 1.69% | 2.25% |
| Total | 2090 | 710 | 100.00% | 64.65% |
| ^a^The rest up to 100% comprised BF (3.50%), AF (0.80%) and FF (0.01%). | | | | |
