# Supplementary material for: Does the Acknowledgement of αS1-Casein Genotype Affect the Estimation of Genetic Parameters and Prediction of Breeding Values for Milk Yield and Composition Quality-Related Traits in Murciano-Granadina?
Source: Animals (Basel). 2019 Sep 13;9(9):679. doi: 10.3390/ani9090679 (PMC6770805; doi:10.3390/ani9090679)
Supplement: Supplementary file 1 [file animals-09-00679-s001.zip › Supplementary Table S3.docx]

**Does the acknowledgement of αS1-casein genotype affect the estimation of genetic parameters and prediction of breeding values for milk yield and composition quality-related traits in Murciano-Granadina?**

**María Gabriela Pizarro Inostroza, Vincenzo Landi, Francisco Javier Navas González, Jose Manuel León Jurado, Amparo Martínez Martínez, Javier Fernández Álvarez and Juan Vicente Delgado Bermejo**

**Supplementary Table S3.** *Testing for normality in milk yield and composition traits in the whole population of Murciano-Granadina goats (N=* *2359479 animals).*

| Trait | Komolgorov-Smirnov | p-value | Kurtosis | Standard error | Skewness | Standard error |
| --- | --- | --- | --- | --- | --- | --- |
| Milk yield (Kg) | 0.062 | 0.000 | 0.873 | 0.002 | 1.218 | 0.003 |
| Fat (Kg) | 0.051 | 0.000 | 0.871 | 0.002 | 1.504 | 0.003 |
| Protein (Kg) | 0.068 | 0.000 | 1.191 | 0.002 | 2.897 | 0.003 |
| Dry matter (Kg) | 0.053 | 0.000 | 0.894 | 0.002 | 1.449 | 0.003 |

|  |  |
| --- | --- |
| SKEWNESS | |
|  | If skewness is less than −1 or greater than +1, the distribution is highly skewed. |
|  | If skewness is between −1 and −½ or between +½ and +1, the distribution is moderately skewed. |
|  | If skewness is between −½ and +½, the distribution is approximately symmetric. |
| Data that are skewed to the right have a long tail that extends to the right, that is a positive Skewness statistic value. In this situation, the mean and the median are both greater than the mode. As a general rule, most of the time for data skewed to the right, the mean will be greater than the median. The situation reverses itself when we deal with data skewed to the left. Data that are skewed to the left have a long tail that extends to the left, that is negatively skewed. In this situation, the mean and the median are both less than the mode. As a general rule, most of the time for data skewed to the left, the mean will be less than the median. | |
|  |  |
| KURTOSIS | |
|  | A normal distribution has kurtosis exactly 3 (excess kurtosis exactly 0). Any distribution with kurtosis ≈3 (excess ≈0) is called mesokurtic. |
|  | A distribution with kurtosis <3 (excess kurtosis <0) is called platykurtic. Compared to a normal distribution, its central peak is lower and broader, and its tails are shorter and thinner. |
|  | A distribution with kurtosis >3 (excess kurtosis >0) is called leptokurtic. Compared to a normal distribution, its central peak is higher and sharper, and its tails are longer and fatter. |
